# Commentary: Cats prefer species-appropriate music

**DOI:** 10.3389/fpsyg.2016.00594

**Published:** 2016-04-28

**Authors:** Cinzia Chiandetti

**Affiliations:** Department of Life Sciences, University of TriesteTrieste, Italy

**Keywords:** consonance, biomusicology, core knowledge, acoustic brick, animate categorization

Whether or not other animal species can appreciate human music is an issue that intrigues thinkers, scientists and artists (as well as pet owners). Yet, to date, despite a rather conspicuous corpus of studies using different investigating strategies with different animal species, no univocal answer does exist. There is contrasting evidence on the effects of music listening on animals' physiology and behavior [for a review Alworth and Buerkle (2013)] and ambiguous results on their appreciation of musical genres. To animals' ears, sometimes silence appears to be more pleasurable than human music (McDermott and Hauser, 2007; Mingle et al., 2014).

At a closer look, the initial question can be reduced to a more substantial one: Why should non-human animals respond to human music? With this query in mind, Snowdon and colleagues composed *ad hoc* music to elicit congruent emotions in cats (Snowdon et al., 2015) and, previously, in cotton-top tamarins (Snowdon and Teie, 2010). By considering ecological and sensorial differences between species, the authors composed music starting from feats of species-specific vocalizations and animals responded to it by emitting emotionally congruent responses. Also, animals' manifestations were stronger and occurred within a shorter delay from the onset of the species-specific musical pieces than when human music was played, thus apparently proving that species-specific music is a far more relevant stimulus for them.

In all musical pieces, Snowdon and colleagues included basic musical feats as modo (major or minor), articulation (legato or staccato) and structure (consonant or dissonant). These feats are universal acoustic bricks that composers regularly use to induce specific feelings in the listeners (Bresin and Friberg, 2011), and that surmount to local variations due to cultural differences (Balkwill and Thompson, 1999).

Such universal acoustic bricks are also recognizable in prosody and similarly affect both human newborns of distant linguistic areas and domesticated animals (Fernald, 1992). Dogs and horses, for instance, respond congruently to verbal commands (McConnnell, 1990) as human infants do, something that has proved to be unrelated to exposure, hence conditioning.

Among these universals, the consonant intervals or structures are as essential as debated. Indeed, on the specific valence of consonant sounds, the results are far from being clear. There are studies showing a preference for this type of sounds in the infants of our species (Zentner and Kagan, 1998), a baby chimp (Sugimoto et al., 2010), and newly-hatched chicks (Chiandetti and Vallortigara, 2011a). Our brain seems to be hard-wired at birth for the appreciation of harmonic tones (Perani et al., 2010), and the babies of deaf people show signs of appreciation as well (Masataka, 2006). There are also studies revisiting the preference in infants (Plantinga and Trehub, 2014) or showing no preference for consonance in monkeys (cotton-top tamarins: McDermott and Hauser, 2004; Campbell's monkeys: Koda et al., 2013). However, note that no research has ever found the opposite pattern of results, i.e., a significant preference for the dissonant version of the stimuli used.

Consonant harmonies were comprised in the calming music composed for cats by Snowdon and colleagues. Hence, such a melodic relation between frequencies is typical in cats' calls, just as it is in general in vocalizations (Schwartz et al., 2003). There are striking examples of consonant intervals in the songs of some species of birds (musician wren: Doolittle and Brumm, 2013; hermit thrush: Doolittle et al., 2014), frogs (Akre et al., 2014), and beyond suspicion mosquitoes that, during their love songs, converge in a harmonic matching (Cator et al., 2009). Furthermore, during a conversation, whenever interlocutors agree, the tonics of the phrasing become consonant (Okada et al., 2012).

Hence, harmonics are naturally present in animals' communication and may well serve to calm species universally, being crucial in affiliative interactions. Then, from the perspective in which consonant harmonies likely represent a pervasive and phylogenetically ancient brick, the question of whether other animal species prefer consonances is a well-posed one. Indeed, a general interest for consonance could be expected in virtue of its ancestral and simple biological function (Bowling and Purves, 2015), which might have been that of an indicator to discriminate animate from inanimate objects (i.e., companions and other animals as distinct from other natural sounds).

A musical feat such as consonance, together with other basic components and related acoustic mechanisms to appreciate them, could complement well-known mechanisms absolving the same purpose in a different sensorial modality: the visual domain. Newborns can visually reason about the basic physical properties of inanimate objects (Baillargeon, 1994) and discriminate objects from agents with internal motivations (Luo and Baillargeon, 2005). Even a precocial species such as the domestic chick represents a world with specific physical (Chiandetti and Vallortigara, 2011b) and psychological laws (Mascalzoni et al., 2010) on the basis of visual characteristics. Several studies now support a Kantian view of the origin of knowledge, posing that a limited set of core knowledge would have been molded by evolution through natural selection and would be endowed in our brains to serve as the basis of our reasoning about physical and social objects, as well as space and number (Spelke, 2000; Carey, 2009; Vallortigara, 2012). This set of core knowledge seems to be largely shared across species (Vallortigara et al., 2010). Along with invariant visual mechanisms, evolution could have shaped acoustic mechanisms to appreciate universal acoustic bricks with the aim of identifying other organisms.

Snowdon et al. (2015) have the merit of having stressed the relevance of the analysis of species-specific sensory signatures to elicit animals' emotional response. However, they based the compositions on universally efficacious musical principles, among which consonance is an example, and that made the pieces attractive for human listeners, too. Cats preferred the melody created for them, but humans liked the music composed for cats. In this sense, their results confirm that there is preference for “musical sounds” in animals, but only limitedly to the interest for features in virtue of their more broad biological and social importance.

Such bricks, typical of living entities, could represent the innate precursor at the basis of the blooming of further musical abilities.

## Author contributions

The author confirms being the sole contributor of this work and approved it for publication.

## Funding

This work was partially supported by a UniTs-FRA2015 grant.

### Conflict of interest statement

The author declares that the research was conducted in the absence of any commercial or financial relationships that could be construed as a potential conflict of interest.
